# Supplementary figures and images for: Mapping of in vivo cleavage sites uncovers a major role for yeast RNase III in regulating protein-coding genes
Source: eLife. 2026 May 18;14:RP106662. doi: 10.7554/eLife.106662 (PMC13183374; doi:10.7554/eLife.106662)

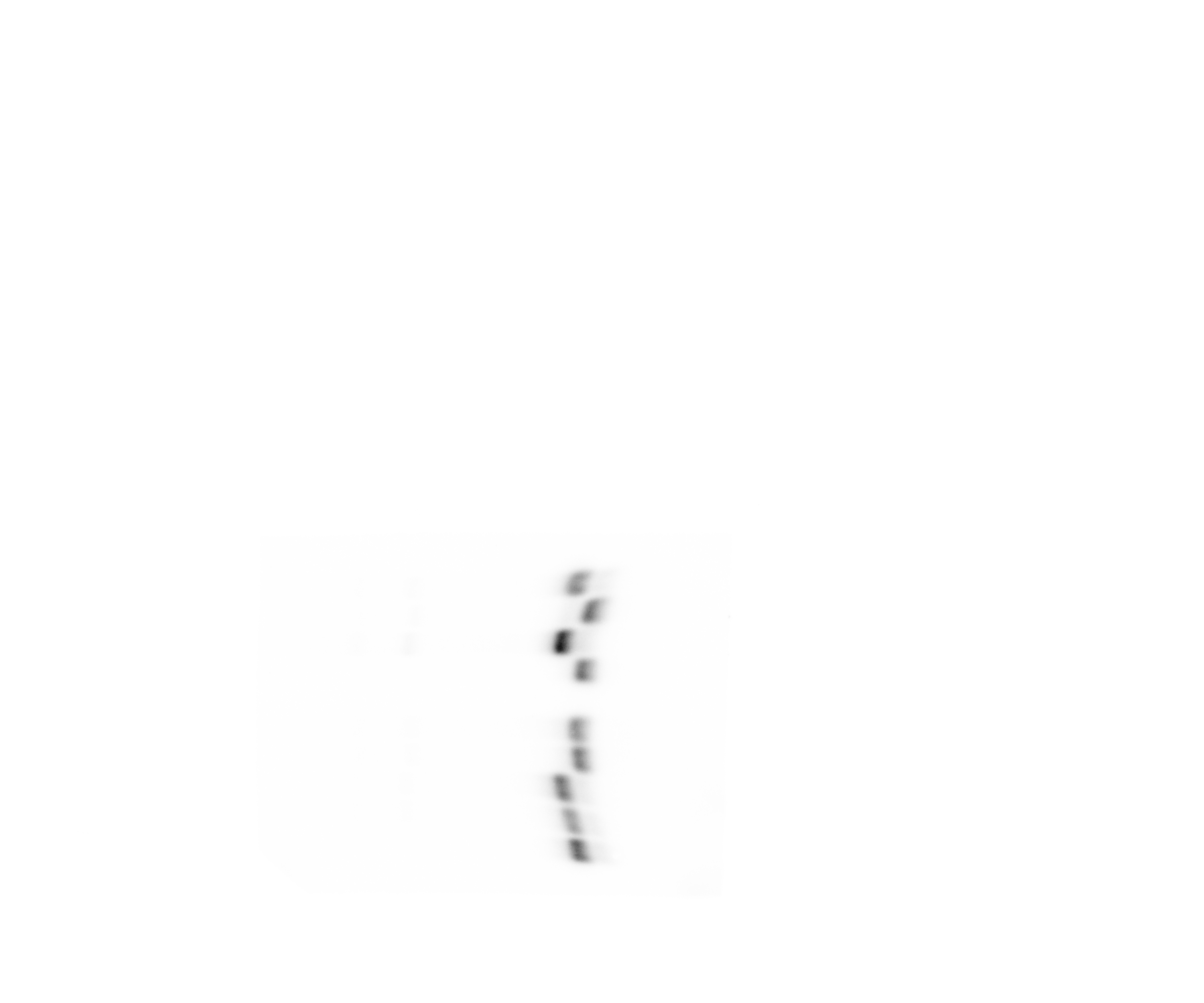

Supplement: Figure 1—source data 1. [file elife-106662-fig1-data1.zip › figure 1E-source data 1.tif]

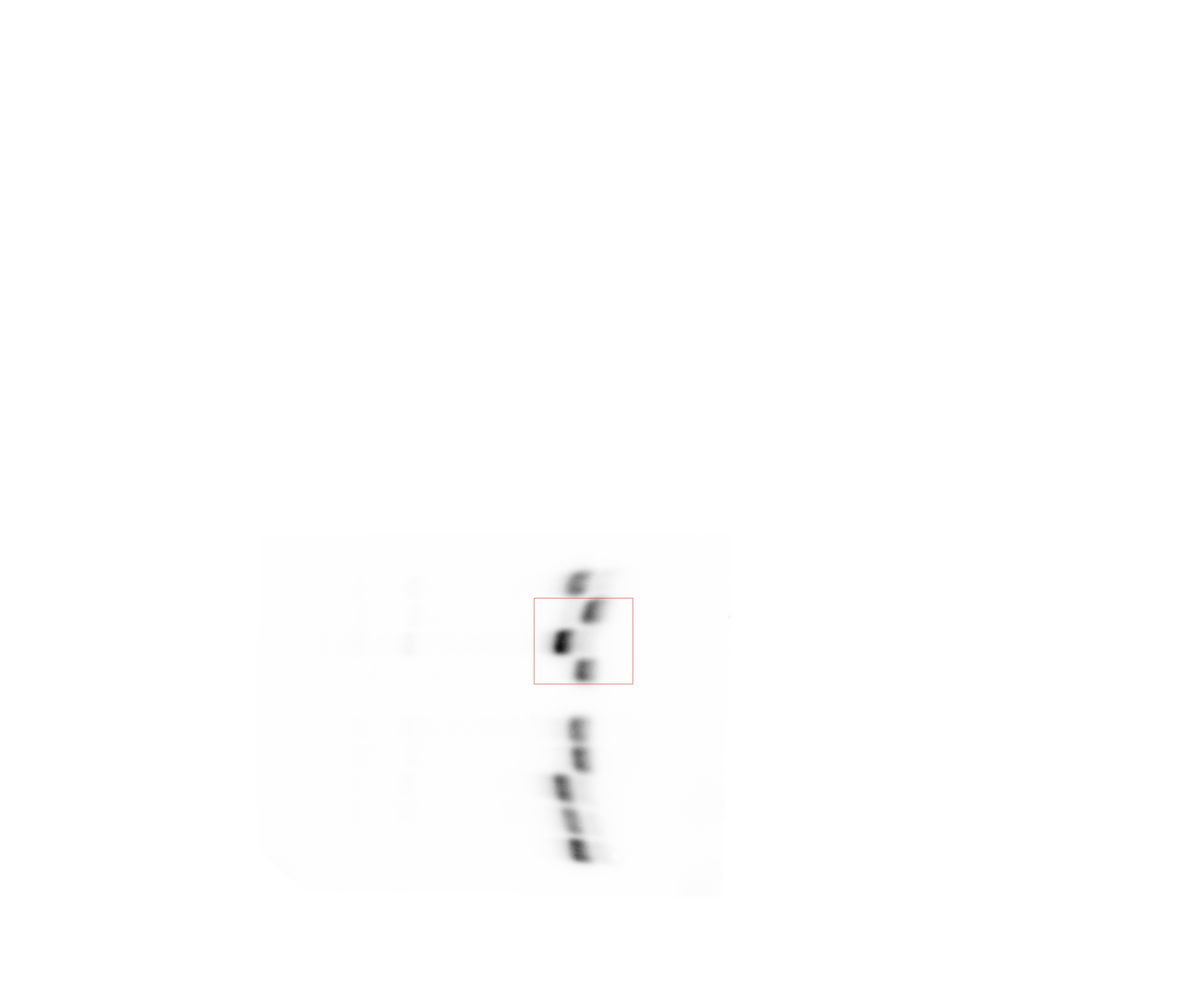

Supplement: Figure 1—source data 2. [file elife-106662-fig1-data2.zip › figure 1E-source data 2.jpg]

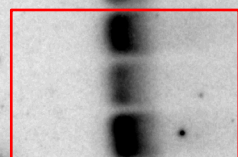

Supplement: Figure 1—figure supplement 3—source data 1. [file elife-106662-fig1-figsupp3-data1.zip › Fig1 Supp 3H-top [GCG1-CHD1] source data 2.pdf]

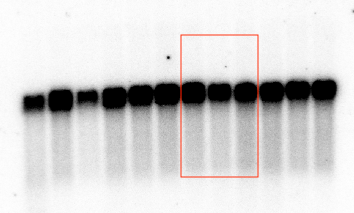

Supplement: Figure 1—figure supplement 3—source data 1. [file elife-106662-fig1-figsupp3-data1.zip › Fig1 Supp Fig 3H-bottom [PGK1] source data 2.png]

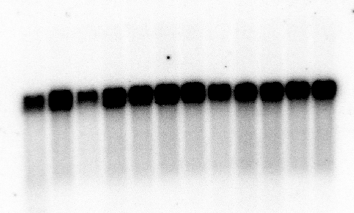

Supplement: Figure 1—figure supplement 3—source data 2. [file elife-106662-fig1-figsupp3-data2.zip › Fig1 Supp Fig 3H-bottom [PGK1] source data 1.png]

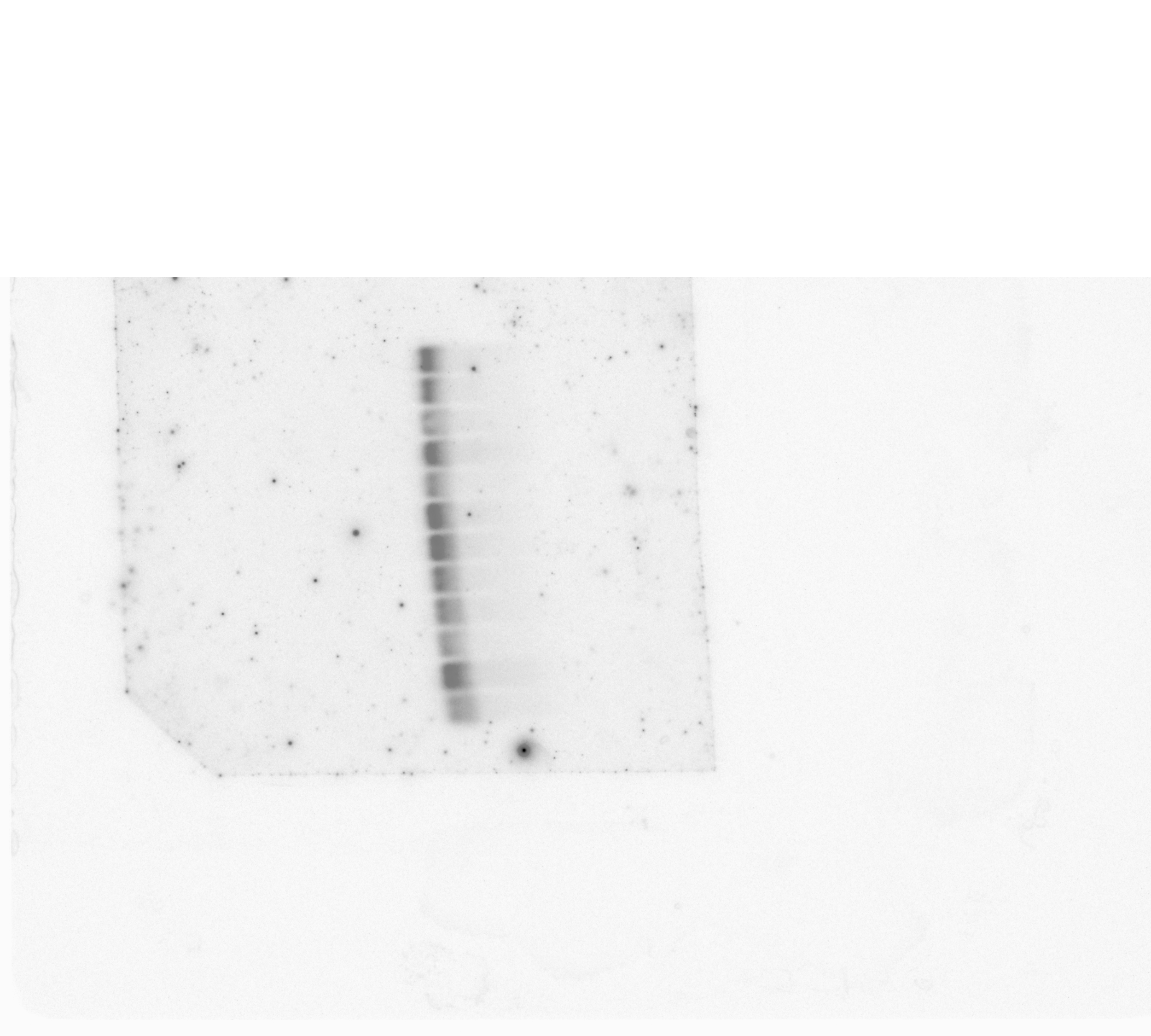

Supplement: Figure 1—figure supplement 3—source data 2. [file elife-106662-fig1-figsupp3-data2.zip › Fig1 Supp 3H-top [GCG1-CHD1] source data 1.tif]

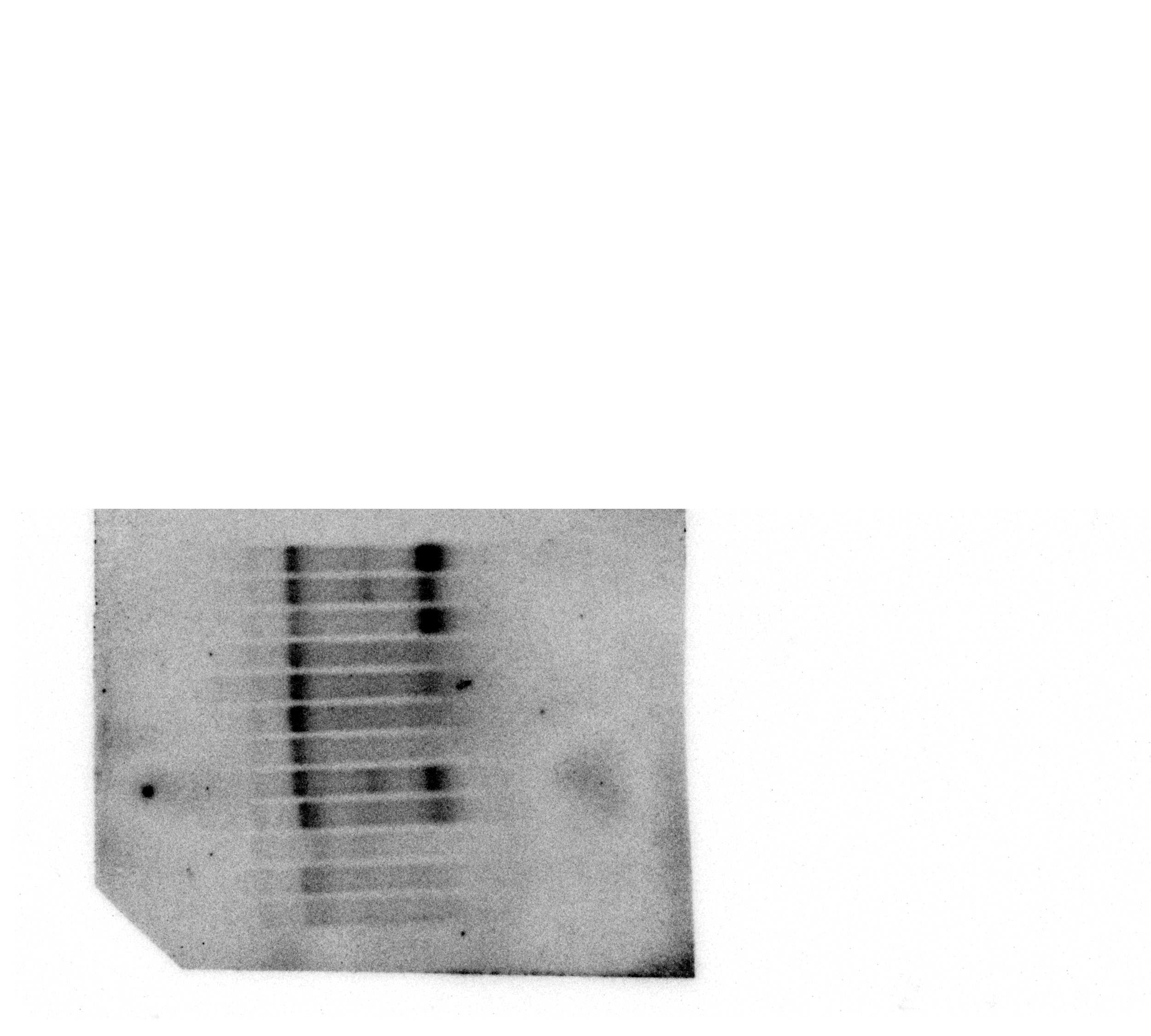

Supplement: Figure 2—source data 1. [file elife-106662-fig2-data1.zip › Figure 2 source data 1/Fig 2E-middle source data 1.tiff]

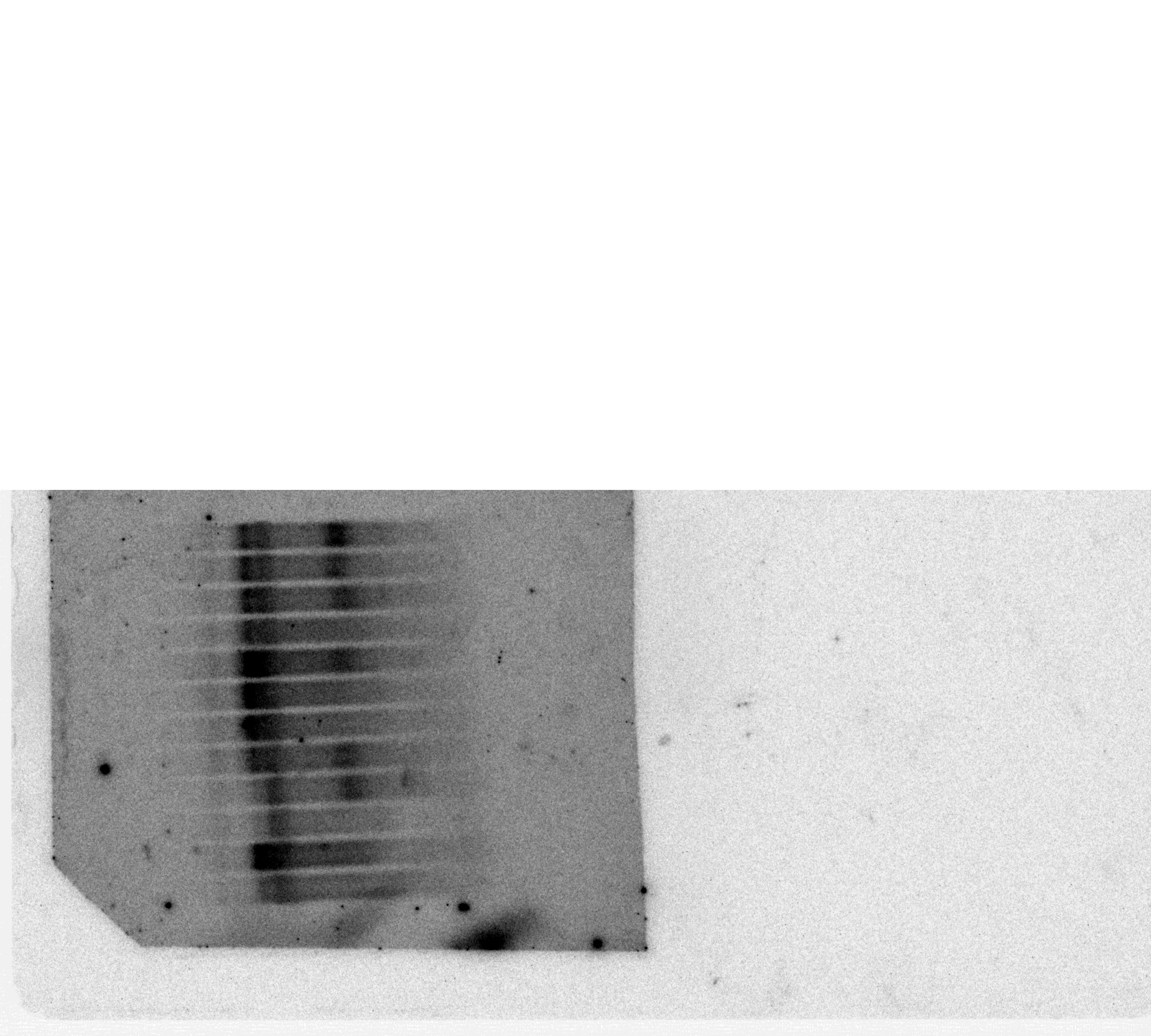

Supplement: Figure 2—source data 1. [file elife-106662-fig2-data1.zip › Figure 2 source data 1/Fig 2E-top-top [BDF2] source data 1.tiff]

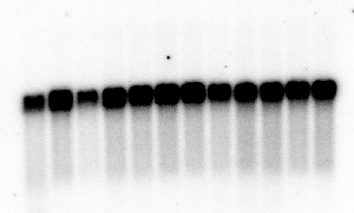

Supplement: Figure 2—source data 1. [file elife-106662-fig2-data1.zip › Figure 2 source data 1/Fig 2E-bottom[PGK1] source data 1.png]

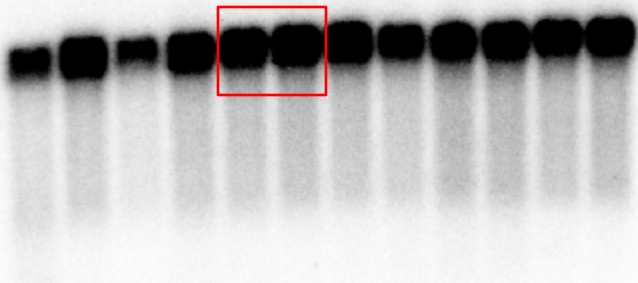

Supplement: Figure 2—source data 2. [file elife-106662-fig2-data2.zip › figure 2 source datat 2/Fig 2E-bottom source data 2.pdf]

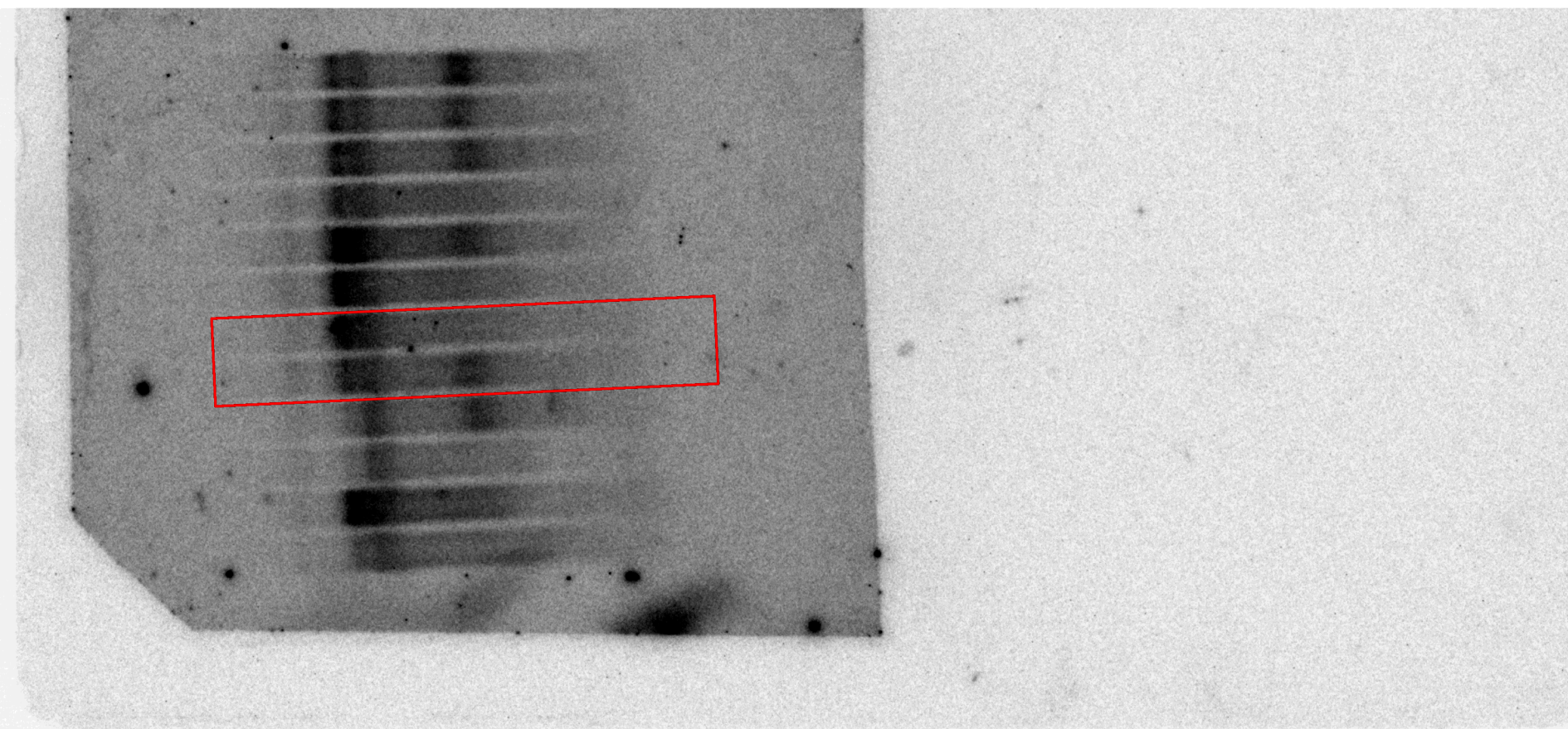

Supplement: Figure 2—source data 2. [file elife-106662-fig2-data2.zip › figure 2 source datat 2/Fig 2E-top source data 2.pdf]

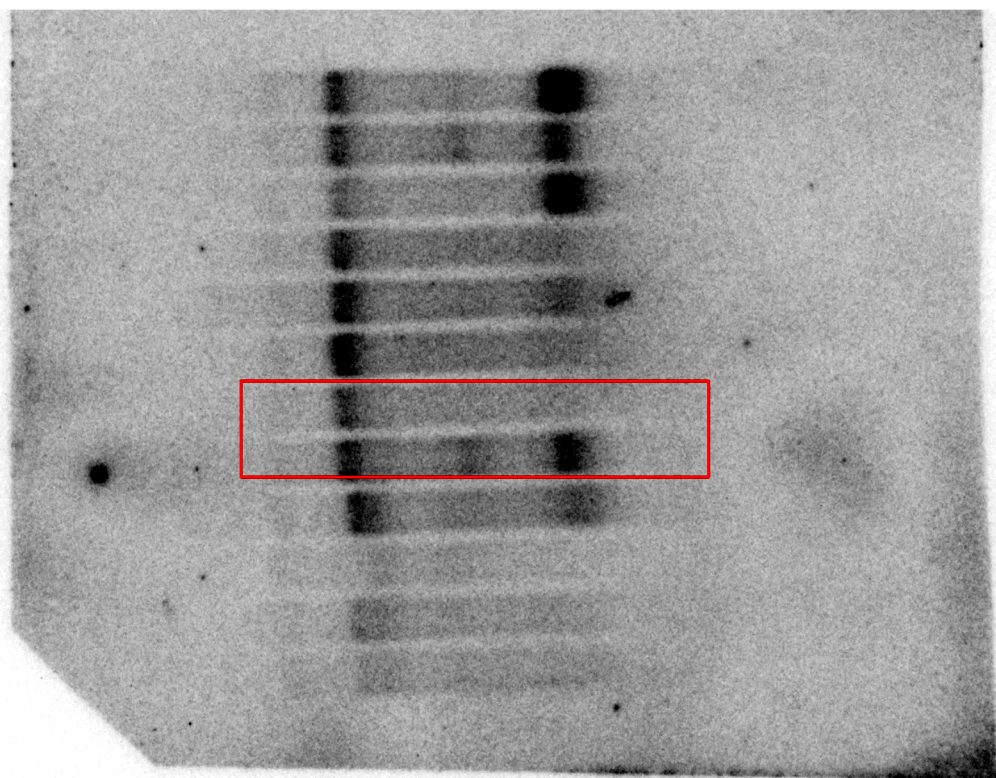

Supplement: Figure 2—source data 2. [file elife-106662-fig2-data2.zip › figure 2 source datat 2/Fig 2E-middle [CAF4] source data 2.pdf]

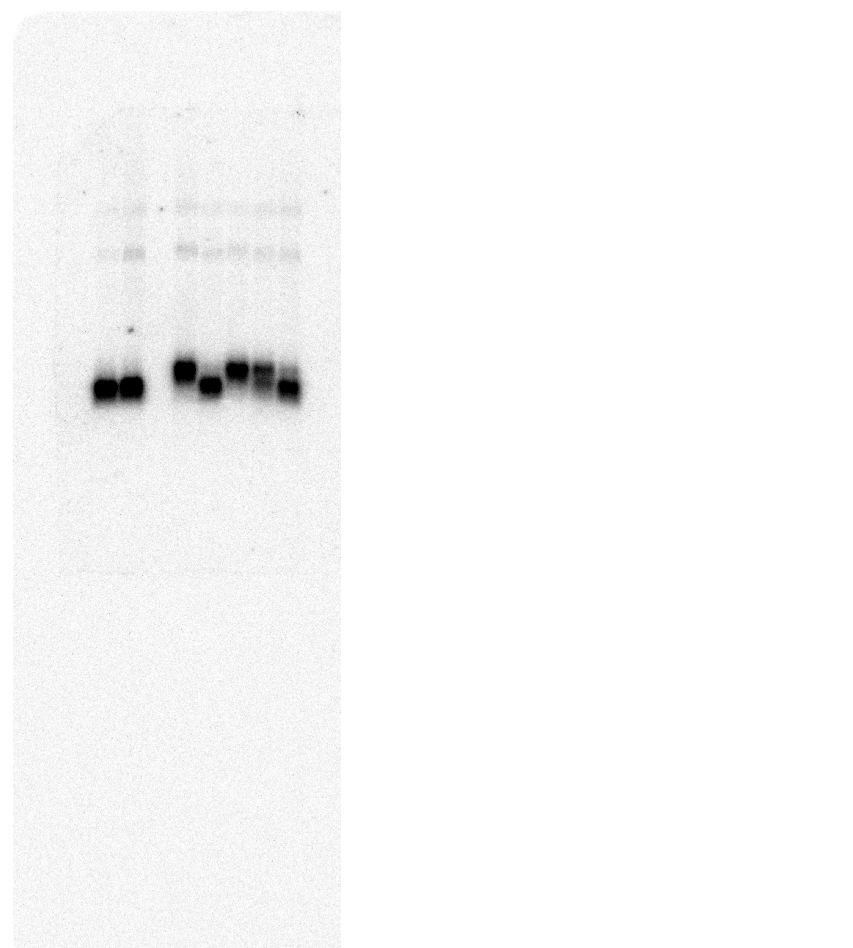

Supplement: Figure 3—source data 1. [file elife-106662-fig3-data1.zip › Fig 3A [SNR83] source data 1.bmp]

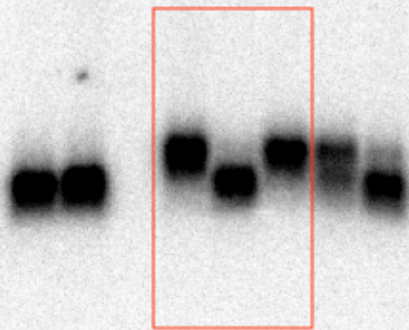

Supplement: Figure 3—source data 2. [file elife-106662-fig3-data2.zip › Fig 3A [SNR83] source data 2.pdf]

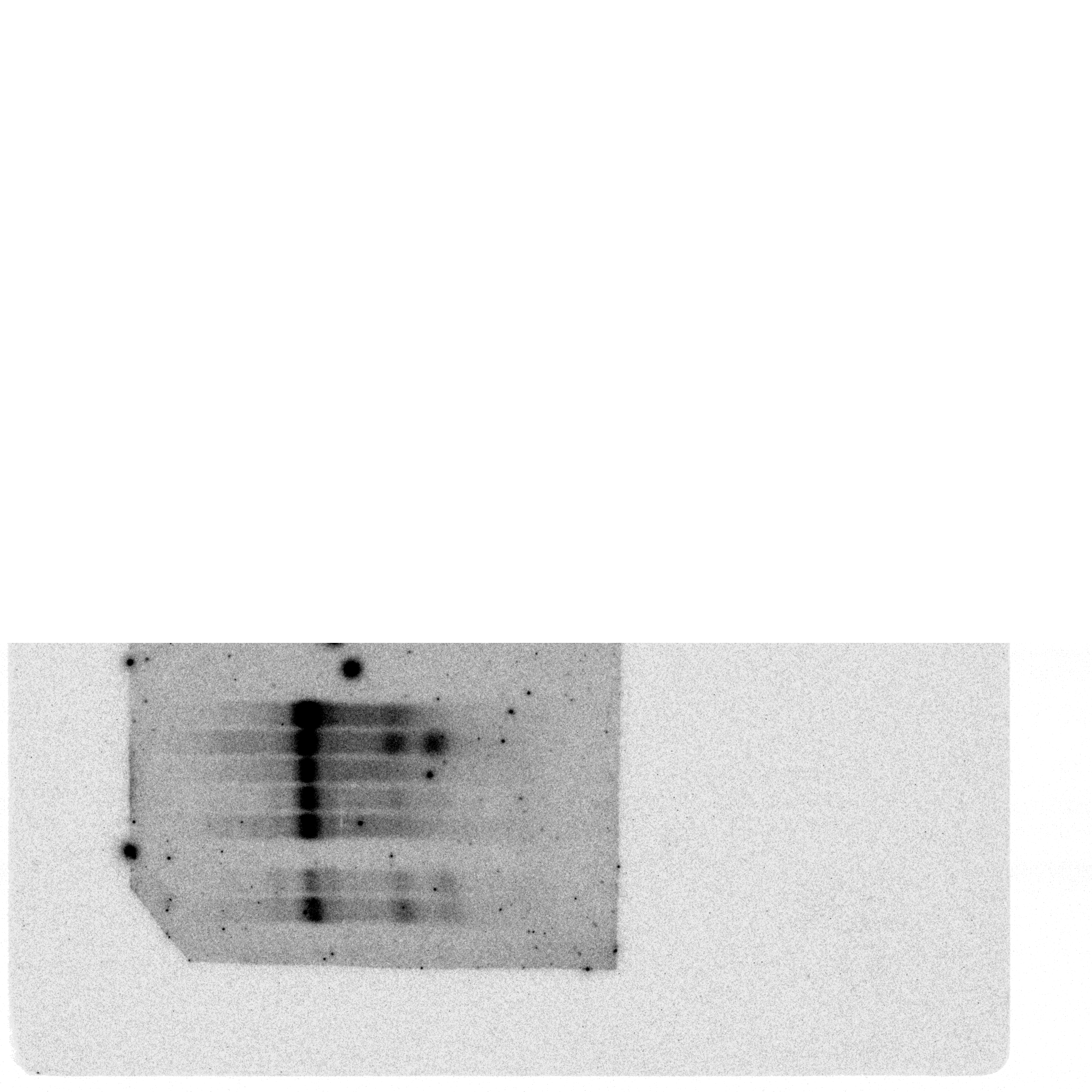

Supplement: Figure 4—source data 1. [file elife-106662-fig4-data1.zip › figure 4 source data 1/Fig 4C-top [BDF2] source data 1.tiff]

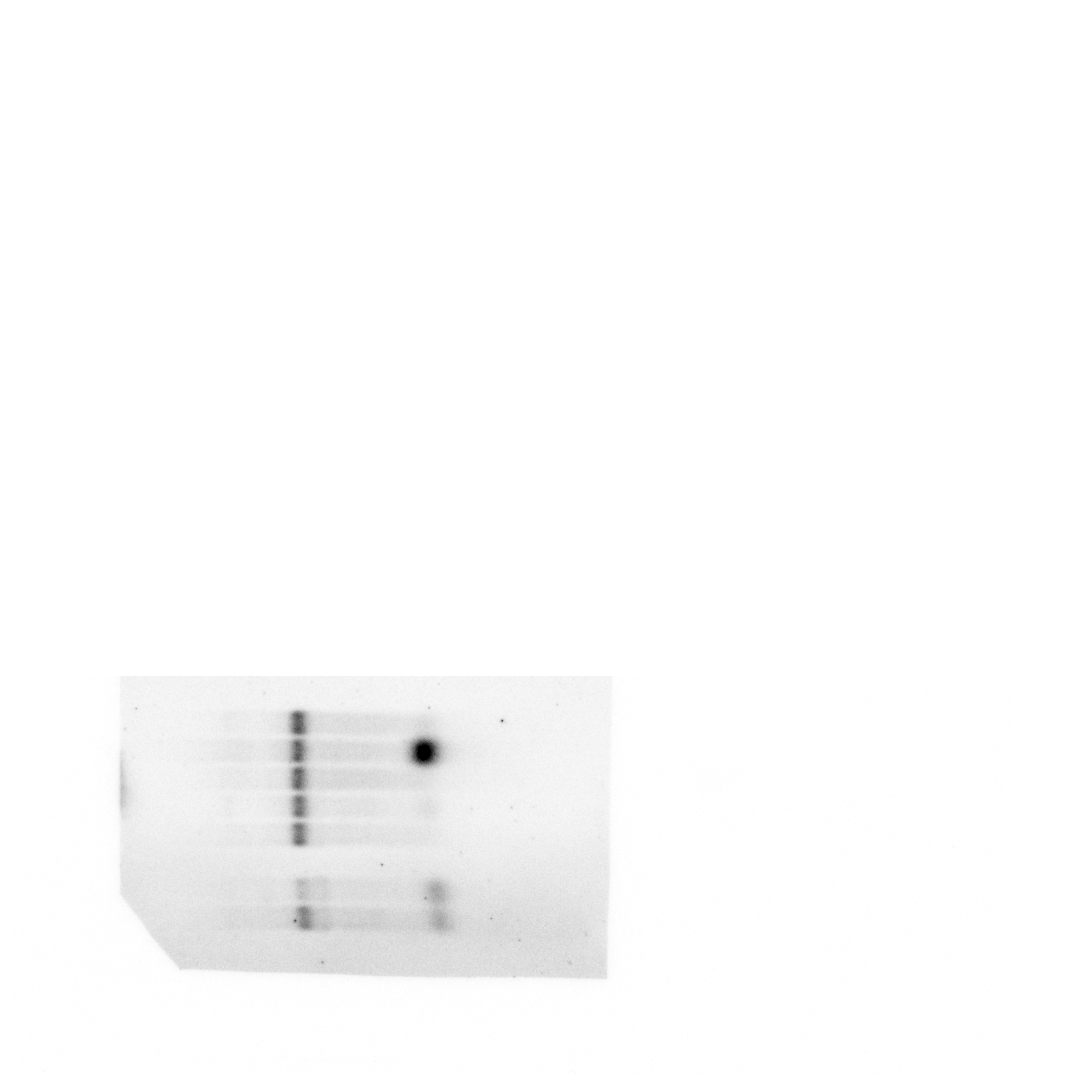

Supplement: Figure 4—source data 1. [file elife-106662-fig4-data1.zip › figure 4 source data 1/Fig 4C-middle [CAF4] source data 1.tiff]

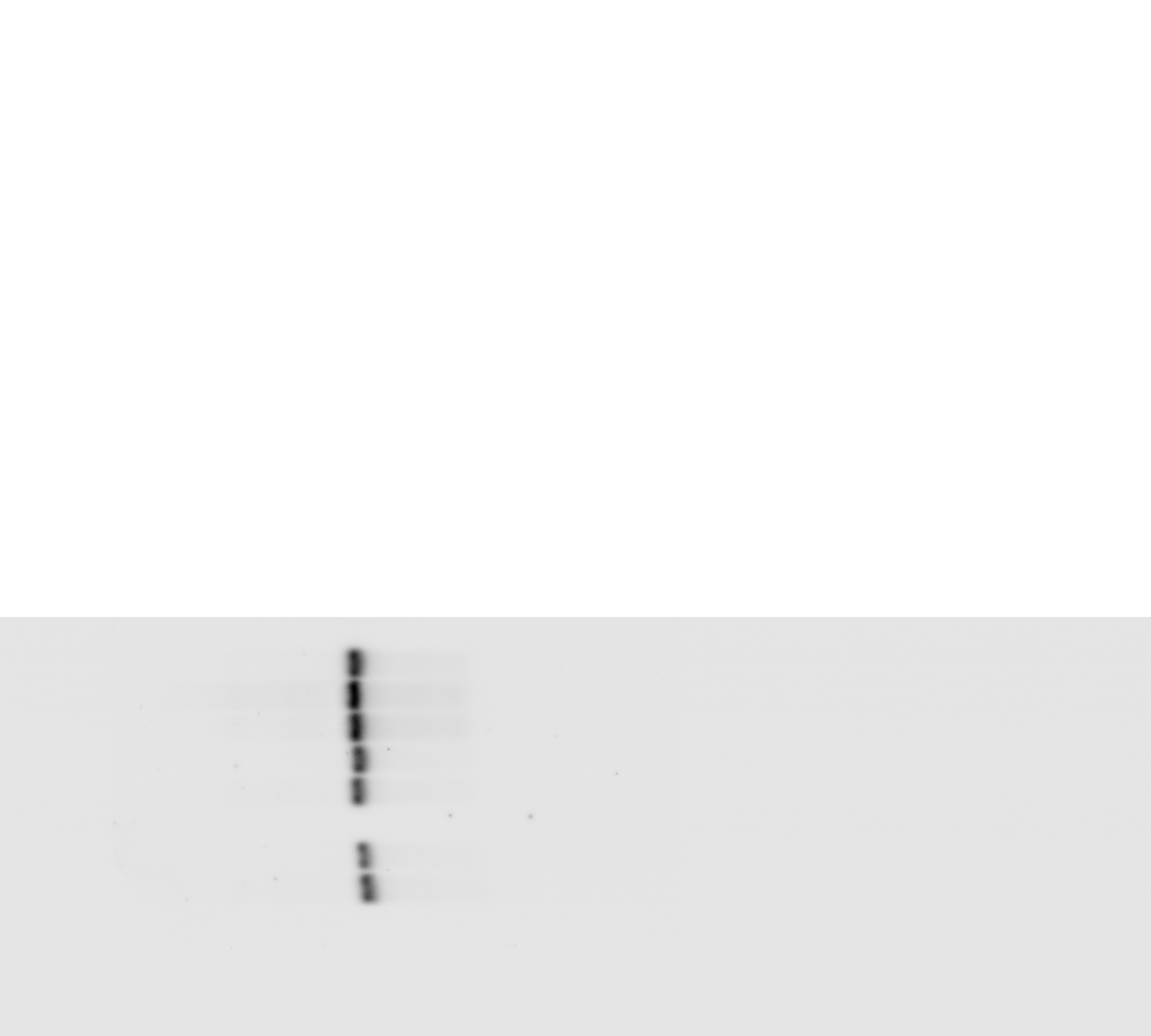

Supplement: Figure 4—source data 1. [file elife-106662-fig4-data1.zip › figure 4 source data 1/Fig 4C-bottom [PGK1] source data 1.tif]

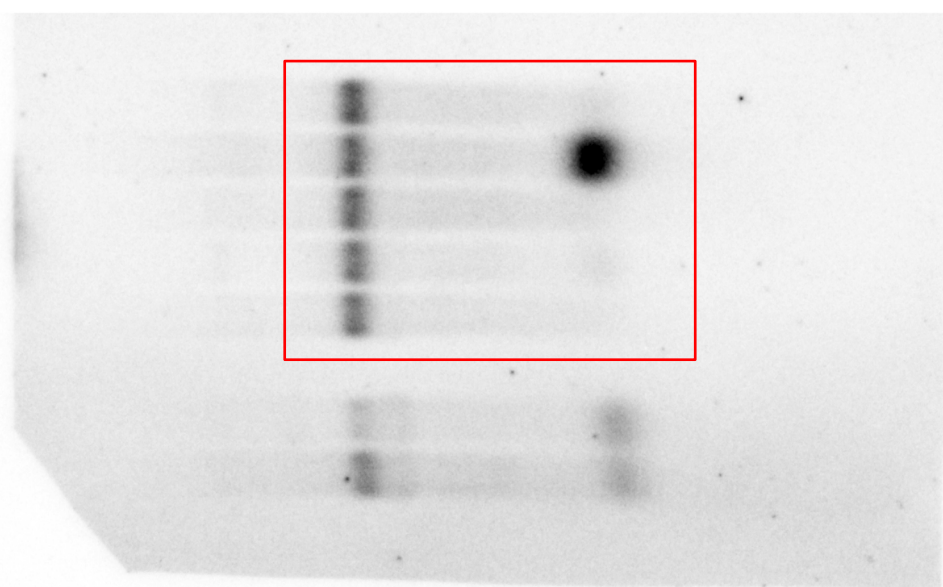

Supplement: Figure 4—source data 2. [file elife-106662-fig4-data2.zip › figure 4 source data 2/Fig 4C-middle [CAF4] source data 2.pdf]

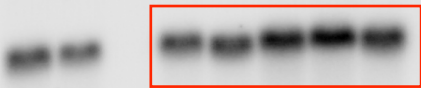

Supplement: Figure 4—source data 2. [file elife-106662-fig4-data2.zip › figure 4 source data 2/Fig 4C-bottom [PGK1] source data 2.pdf]

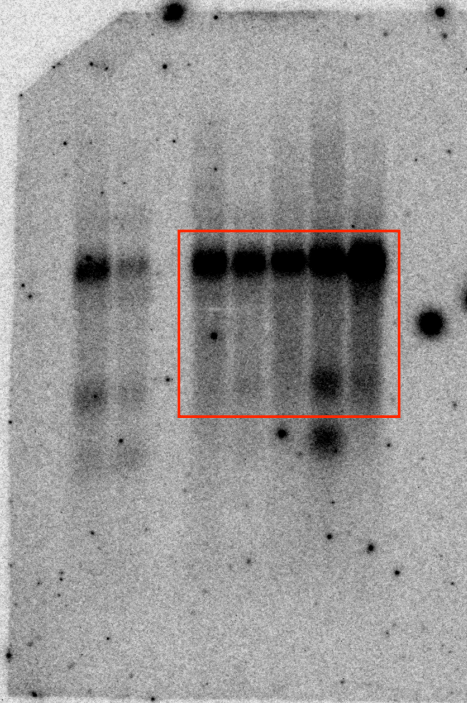

Supplement: Figure 4—source data 2. [file elife-106662-fig4-data2.zip › figure 4 source data 2/Fig 4C-top [BDF2] source data 2.pdf]

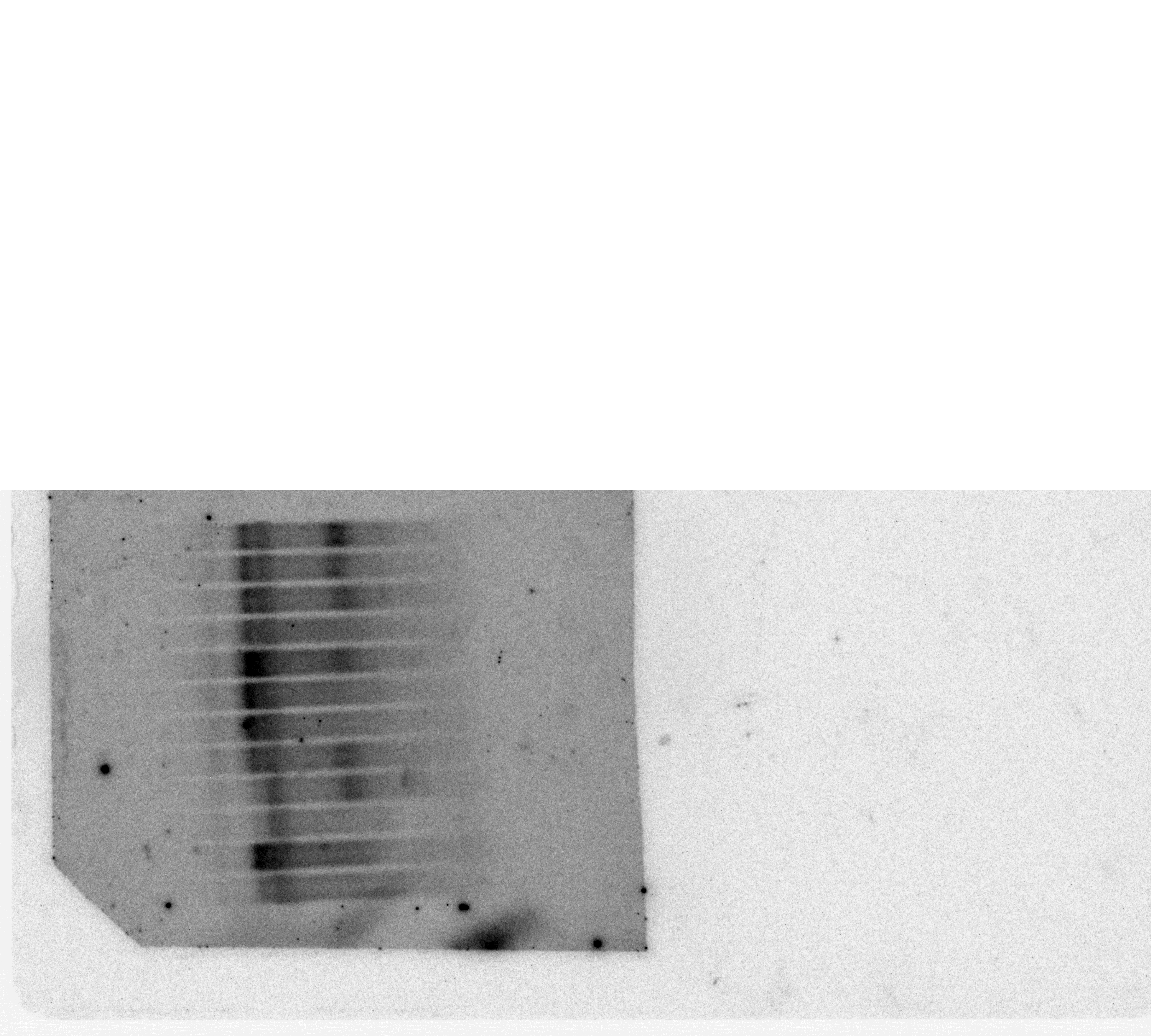

Supplement: Figure 5—source data 1. [file elife-106662-fig5-data1.zip › fugure 5 source data 1/Fig 5B-top [BDF2] soucre data 1.tiff]

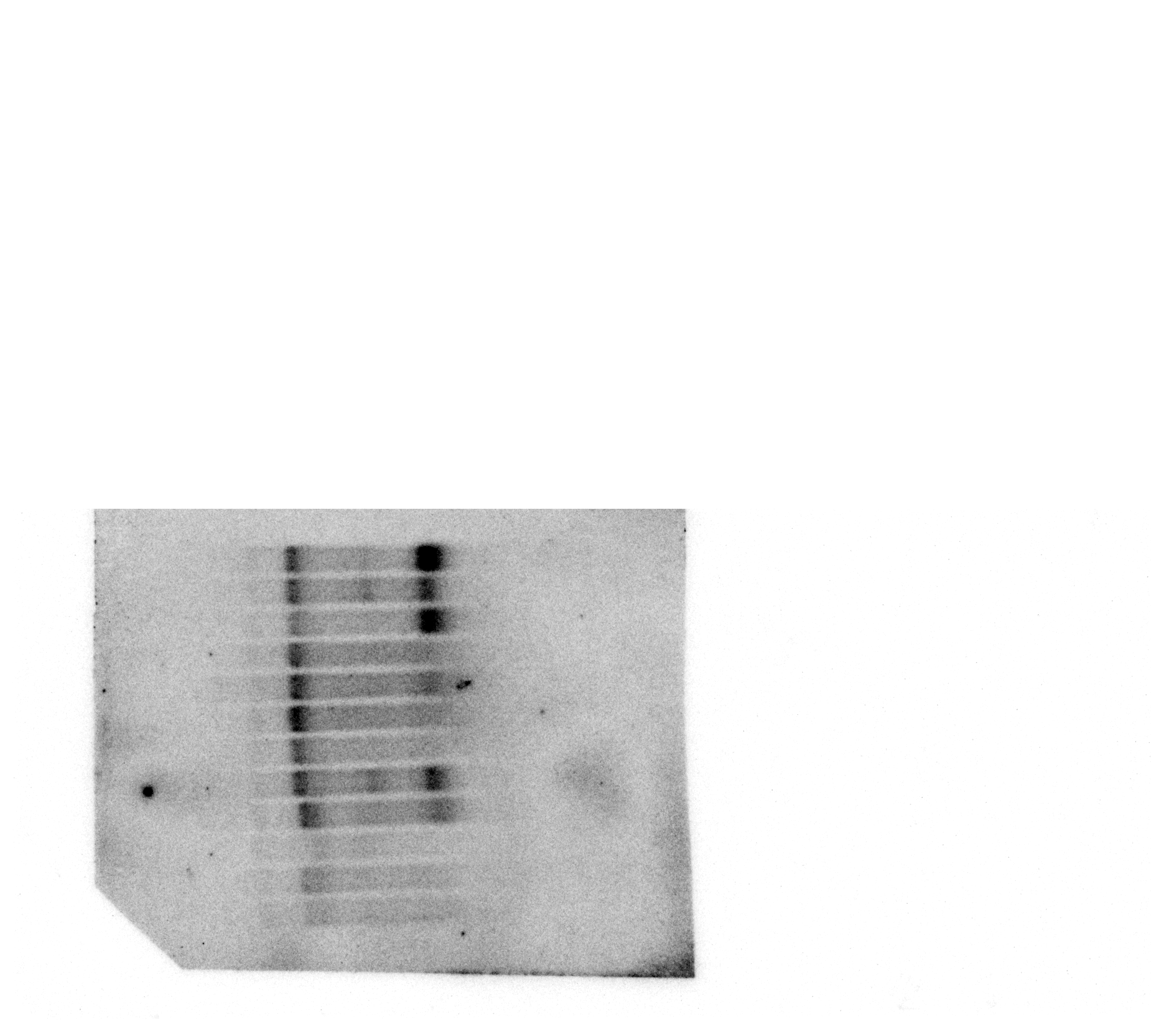

Supplement: Figure 5—source data 1. [file elife-106662-fig5-data1.zip › fugure 5 source data 1/Fig 5B-middle [CAF4] source data 1.tiff]

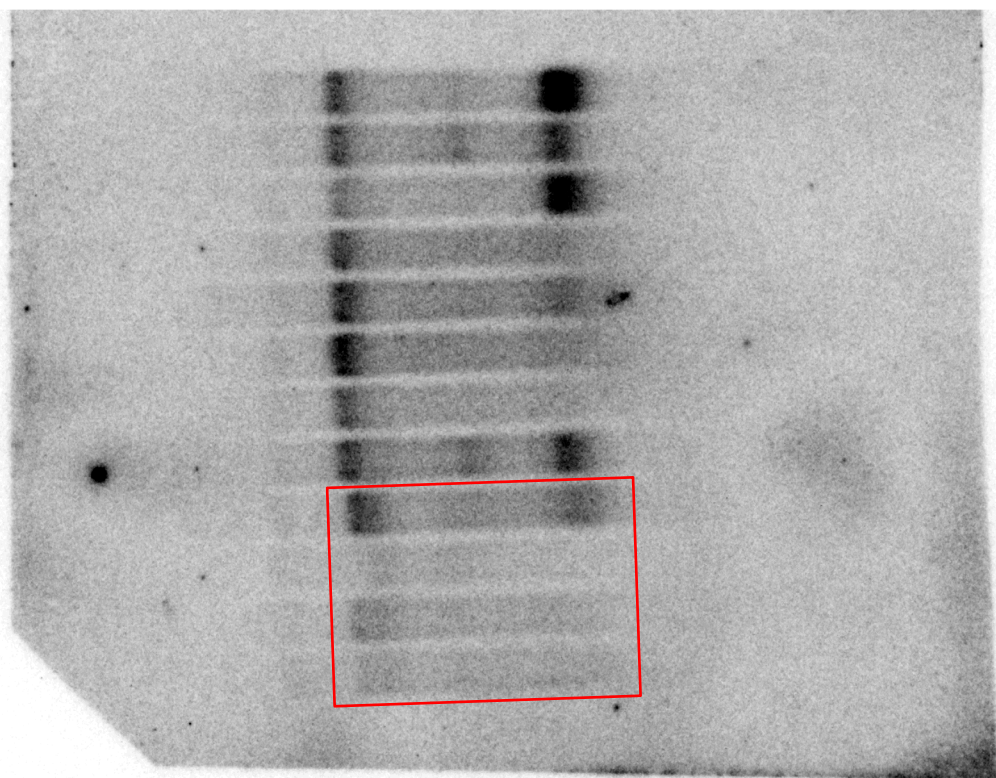

Supplement: Figure 5—source data 2. [file elife-106662-fig5-data2.zip › fugure 5 source data 2/Fig 5B-middle [CAF4] source data 2.pdf]

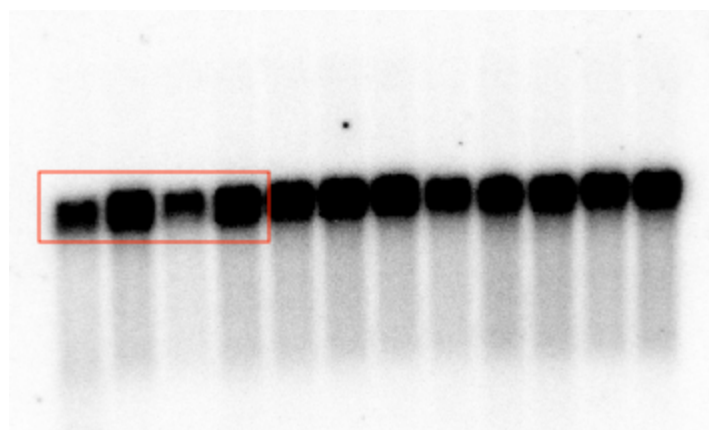

Supplement: Figure 5—source data 2. [file elife-106662-fig5-data2.zip › fugure 5 source data 2/Fig 5B-bottom PGK1] source data 2.pdf]

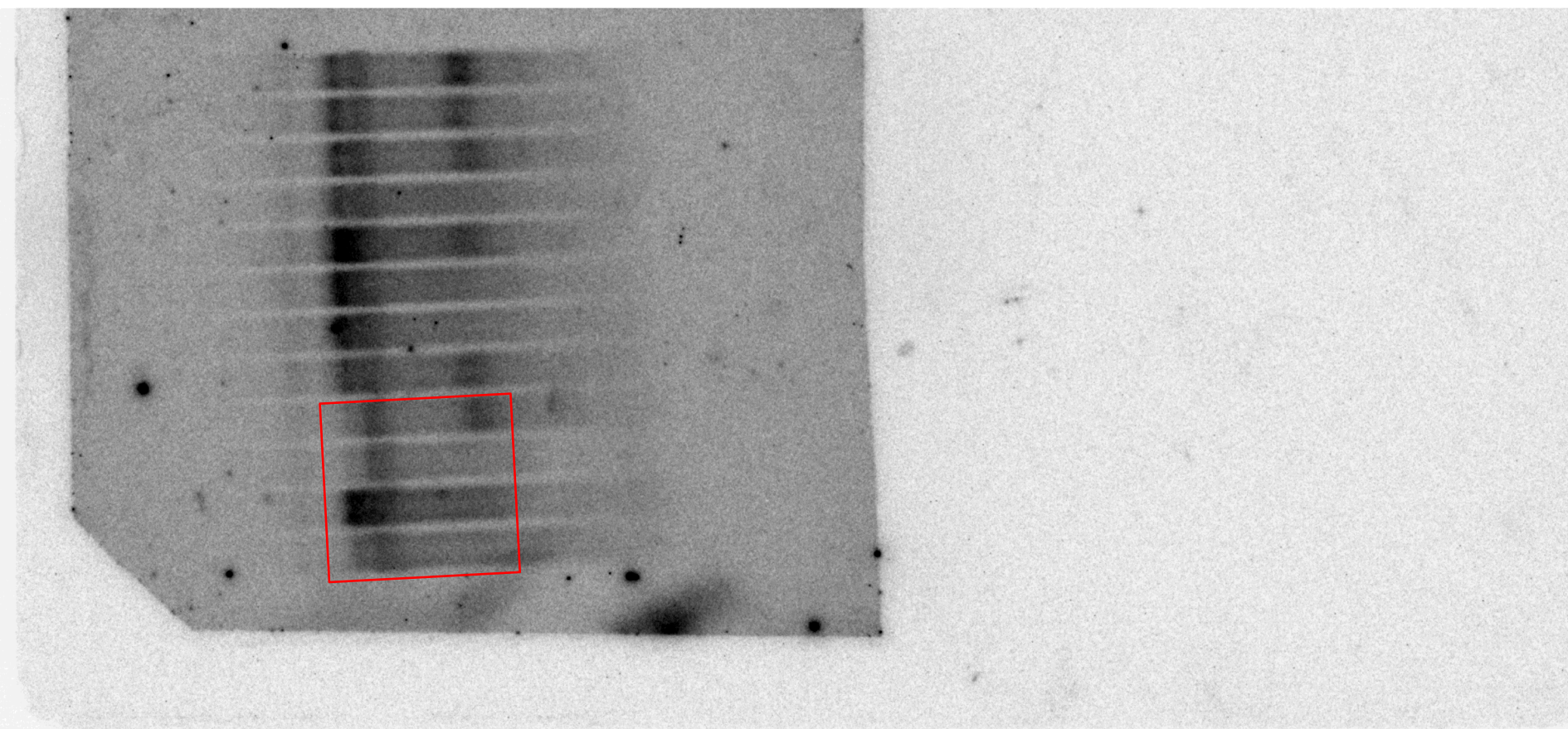

Supplement: Figure 5—source data 2. [file elife-106662-fig5-data2.zip › fugure 5 source data 2/Fig 5B-top [BDF2] source data 2.pdf]
