# Supplementary material for: Mapping of in vivo cleavage sites uncovers a major role for yeast RNase III in regulating protein-coding genes
Source: eLife. 2026 May 18;14:RP106662. doi: 10.7554/eLife.106662 (PMC13183374; doi:10.7554/eLife.106662)
Supplement: Supplementary file 2. [file elife-106662-supp2.docx]

**Table S2:** List of plasmids used in this study:

| **Plasmid** | **Description** | **Source** |
| --- | --- | --- |
| pAG32 | hphMX4, conferring resistance to Hygromycin B | Goldstein & McCusker, 1999 |
| pRS415 | CEN empty vector with *LEU2* gene | Sikorski & Hieter, 1989 |
| pAV1595 | CEN pRS315 containing GFP under native *RNT1* promoter, *LEU2* | Catala et al., 2004 |
| pAV1596 | CEN pRS315 containing *RNT1* CDS (residues 1-471) with N-terminal GFP tag, under native *RNT1* promoter, *LEU2* | Catala et al., 2004 |
| pAV1597 | CEN pRS315 containing *RNT1* CDS (residues 1-471) with *D245R* mutation and N-terminal GFP tag, under native *RNT1* promoter, *LEU2* | Catala et al., 2004 |
| pAV412 | CEN pRS315 containing C-terminal-truncated *RNT1* CDS (residues 1-463) with N-terminal GFP tag, under native *RNT1* promoter, *LEU2* | Catala et al., 2004 |
| pAV413 | CEN pRS315 containing *RNT1* CDS (residues 1-471) with *K45I* mutation and N-terminal GFP tag, under native *RNT1* promoter, *LEU2* | Catala et al., 2004 |
| p425GPD | 2µ p425GPD empty vector, LEU2 | Mumberg et al., 1995 |
| pAV2084 | 2µ p425GPD YDR514C under GPD promoter, LEU2 | This study |
| pAV2085 | 2µ p425GPD YDR514C-SL* under GPD promoter, LEU2 | This study |
