# Supplementary material for: Mapping of in vivo cleavage sites uncovers a major role for yeast RNase III in regulating protein-coding genes
Source: eLife. 2026 May 18;14:RP106662. doi: 10.7554/eLife.106662 (PMC13183374; doi:10.7554/eLife.106662)
Supplement: Supplementary file 3. [file elife-106662-supp3.docx]

**Table S3:** List of yeast strains used in this study:

| **Name** | **Genotype** | **Source** |
| --- | --- | --- |
| BY4742 | *MATα*, *ura3Δ-0*, *leu2Δ-0*, *his3Δ-1*, *lys2Δ-0*, *MET15* | Baker Brachmann et al., 1998 |
| yAV1085 | *MATα*, *ura3Δ-0*, *leu2Δ-0*, *his3Δ-1*, *lys2Δ-0*, *met15Δ-0*, *rnt1Δ::NEO* | This study |
| yAV4514 | *MATa*, *ura3Δ-0*, *leu2Δ-0*, *his3Δ-1*, *lys2Δ-0*, *met15Δ-0*, *rnt1Δ::HYG* | This study |
| N.A. | *MATa, ura3Δ-0, leu2Δ-0, his3Δ-200, lys2Δ-0, rnt1Δ::HIS3* | Catala et al., 2012 |
| yAV5012 | *MATα*, *ura3Δ-0*, *leu2Δ-0*, *his3Δ-1*, *lys2Δ-0*, *met15Δ-0*, *rnt1Δ::NEO [LEU2]* | This study |
| yAV4419 | *MATα*, *ura3Δ-0*, *leu2Δ-0*, *his3Δ-1*, *lys2Δ-0*, *met15Δ-0*, *rnt1Δ::NEO [GFP, RNT1, LEU2]* | This study |
| yAV4421 | *MATα*, *ura3Δ-0*, *leu2Δ-0*, *his3Δ-1*, *lys2Δ-0*, *met15Δ-0*, *rnt1Δ::NEO [GFP, rnt1-ΔNLS, LEU2]* | This study |
| yAV4422 | *MATα*, *ura3Δ-0*, *leu2Δ-0*, *his3Δ-1*, *lys2Δ-0*, *met15Δ-0*, *rnt1Δ::NEO [GFP,* *rnt1-K45I, LEU2]* | This study |
| yAV3484 | *MATa*, *ura3Δ-0*, *leu2Δ-0*, *his3Δ-1*, *LYS2*, *MET15*, *rat1-ts::URA3*, *can1 Δ::LEU2*, *MFApr-HIS3* | Kofoed et al., 2015 |
| yAV3248 | *MATα*, *ura3Δ-0*, *leu2Δ-0*, *his3Δ-1*, *lys2Δ-0*, *MET15*, *xrn1Δ::HYG* | Hurtig et al., 2021 |
| yAV4752 | *MATα*, *ura3Δ-0*, *leu2Δ-0*, *his3Δ-1*, *LYS2*, *MET15*, *puf4Δ::NEO* | This study |
| yAV4754 | *MATα*, *ura3Δ-0*, *leu2Δ-0*, *his3Δ-1*, *LYS2*, *MET15*, *ydr514cΔ::NEO* | This study |
| yAV4212 | *MATα*, *ura3Δ-0*, *leu2Δ-0*, *his3Δ-1*, *lys2Δ-0*, *met15Δ-0*, *rat1-ts::URA3*, *MFApr-HIS3*, *xrn1Δ::HYG* | This study |
| yAV4954 | *MATα*, *ura3Δ-0*, *leu2Δ-0*, *his3Δ-1*, *LYS2*, *met15Δ-0*, *rnt1Δ::HYG*, *puf4Δ::NEO* | This study |
| yAV4956 | *MATα*, *ura3Δ-0*, *leu2Δ-0*, *his3Δ-1*, *lys2Δ-0*, *met15Δ-0*, *rnt1Δ::HYG*, *ydr514cΔ::NEO* | This study |
| yAV4054 | *MATα*, *ura3Δ-0*, *leu2Δ-0*, *his3Δ-1*, *lys2Δ-0*, *met15Δ-0*, *rnt1Δ::NEO*, *rat1-ts::URA3*, *MFApr-HIS3*, *xrn1Δ::HYG* | This study |
| yAV4070 | *MATα*, *ura3Δ-0*, *leu2Δ-0*, *his3Δ-1*, *lys2Δ-0*, *met15Δ-0*, *rnt1Δ::NEO*, *rat1-ts::URA3*, *xrn1Δ::HYG [GFP, LEU2]* | This study |
| yAV4071 | *MATα*, *ura3Δ-0*, *leu2Δ-0*, *his3Δ-1*, *lys2Δ-0*, *met15Δ-0*, *rnt1Δ::NEO*, *rat1-ts::URA3*, *xrn1Δ::HYG [GFP, RNT1, LEU2]* | This study |
| yAV4072 | *MATα*, *ura3Δ-0*, *leu2Δ-0*, *his3Δ-1*, *lys2Δ-0*, *met15Δ-0*, *rnt1Δ::NEO*, *rat1-ts::URA3*, *xrn1Δ::HYG [GFP, rnt1-D245R, LEU2]* | This study |
| yAV4193 | *MATα*, *ura3Δ-0*, *leu2Δ-0*, *his3Δ-1*, *lys2Δ-0*, *met15Δ-0*, *rnt1Δ::NEO*, *rat1-ts::URA3*, *xrn1Δ::HYG [GFP, rnt1-ΔNLS, LEU2]* | This study |
| yAV4194 | *MATα*, *ura3Δ-0*, *leu2Δ-0*, *his3Δ-1*, *lys2Δ-0*, *met15Δ-0*, *rnt1Δ::NEO*, *rat1-ts::URA3*, *xrn1Δ::HYG [GFP,* *rnt1-K45I, LEU2]* | This study |
